# Biomechanical comparison between solid and cannulated intramedullary devices for midshaft clavicle fixation

**DOI:** 10.1186/s12891-019-2560-x

**Published:** 2019-04-25

**Authors:** Sheng-Hao Wang, Hsiu-Jen Lin, Hsain-Chung Shen, Ru-Yu Pan, Jui-Jung Yang

**Affiliations:** 1 Department of Orthopaedics, Tri-Service General Hospital, National Defense Medical Center, No. 325, Cheng-Kung Road Section 2, Taipei, 114 Taiwan, Republic of China; 2 Graduate Institute of Medical Science, National Defense Medical Center, Taipei, Taiwan; 3 Department of Mechanical Engineering, National Taipei University of Technology, Taipei, Taiwan

**Keywords:** Intramedullary device, Midshaft clavicle fracture, Cannulated screw

## Abstract

**Background:**

A method of closed reduction and internal fixation with cannulated screws was proposed as a surgical treatment of midshaft clavicle fractures. However, there are no mechanical studies about the cannulated screw used in the fixation of midshaft clavicle fracture. We conducted this study to compare the construct bending stiffness of a fixation midshaft clavicle fracture with a Knowles pin, cannulated screw and reconstruction plate. In addition, purchase lengths of both intramedullary devices were measured.

**Methods:**

After transverse osteotomy over the midpoint for fracture simulation, eighteen synthetic clavicles were assigned to 3 groups and fixed with reconstruction plate, Knowles pin or cannulated screw. Purchase length was defined as the engaged length of the intramedullary portion of the two intramedullary devices Stiffness, yield load and maximum load of the cantilever bending test were calculated of each tested synthetic bones.

**Results:**

The Knowles pin group had a significantly longer average intramedullary purchase length compared with that of the cannulated screw group. The construct stiffness in the reconstruction plate group (5.6 ± 0.9 N/mm) was higher than that of the intramedullary devices; the Knowles pin group (3.1 ± 0.6 N/mm) provided a greater construct stiffness than did the cannulated screw group (1.7 ± 0.4 N/mm) (*p* = 0.007). The cannulated screw group had the lowest yield and maximum load compared with the reconstruction plate and Knowles pin groups. Both the reconstruction plate and Knowles pin failed at the implant-bone interface. However, the cannulated screw group failed at the osteotomy site with broken implants.

**Conclusion:**

This study suggests that fixation of midshaft clavicle fractures with cannulated screws may lead to early failure due to inadequate mechanical strength. Ideal intramedullary clavicle devices should supply adequate intramedullary purchase lengths and mechanical strength.

## Background

Midshaft fractures account for 80% of clavicle fractures [1]. Traditionally, even displaced fractures were managed with conservative treatments based on the satisfactory results of several previous studies [2]. However, recent studies have reported unsatisfactory results of conservative treatments [3, 4]. A more aggressive attitude with surgical treatment had been suggested [5].

Surgical implants of midshaft clavicle fixation can be summarized into two types: plates and intramedullary fixation. To reduce the incidence of wound infection and fracture nonunion, a closed or minimally invasive method using intramedullary fixation with cannulated screws had been proposed [6–9]. However, there are no mechanical studies on the use of cannulated screws in the fixation of midshaft clavicle fractures. The hollow structure of a cannulated screw may weaken the mechanical strength of the implant. The larger diameter and shorter intramedullary purchase length of the cannulated screw, compared with other intramedullary devices, may be a drawback [10].

This study evaluated the construct bending stiffness of cannulated screws used to repair a simulated midshaft fracture of synthetic clavicles under a cantilever bending test. It was hypothesized that the mechanical strength of a cannulated screw would be at least equal to that provided by a Knowles pin or a reconstruction plate.

## Methods

### Specimens

Eighteen fourth-generation synthetic clavicles (Pacific Research Laboratories, Vashon Island, WA, USA) were assigned to three groups according to fixation method (reconstruction plate, Knowles pin, and cannulated screw). Previous studies have reported the comparable failure modes, stiffness, and strength of composite bones to cadaveric bones, but without the anatomical variability present in cadaveric models [11, 12]. Transverse osteotomy over the midpoint was performed with an oscillating saw to simulate a midshaft clavicle fracture. Then, the fragments were fixed anatomically by either extramedullary (reconstruction plate) or intramedullary (Knowles pin or cannulated screw) fixation.

### Instrumentation

For the reconstruction plate group, a 6-hole, 3.5 mm stainless reconstruction plate (Smith & Nephew, Tuttlingen, Germany) was contoured to the superior surface of the synthetic bone. The AO (Arbeitsgemeinschaft für Osteosynthesefragen) technique was used to place 3.5-mm cortical, fully threaded screws; holes were drilled, tapped, and measured prior to screw selection.

**Fig. 1:**
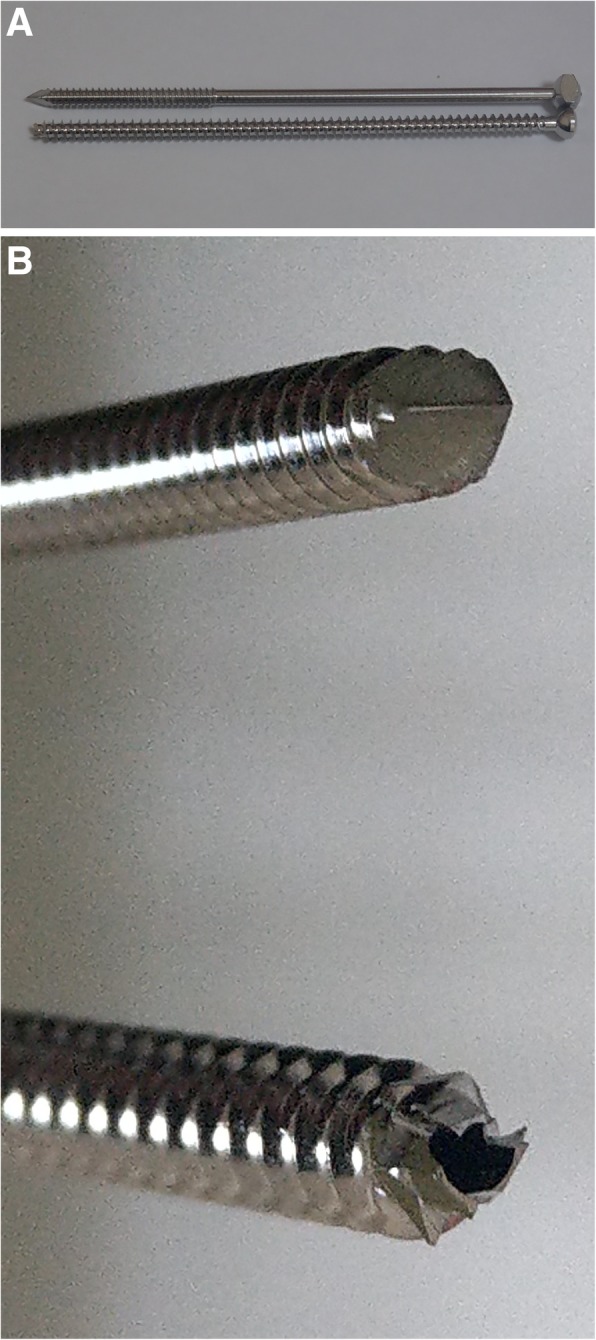
Photographs of 3.9 mm diameter Knowles pin (top) and 4.5 mm diameter cannulated screw (bottom). **a** side view and (**b**) front view

**Fig. 2:**
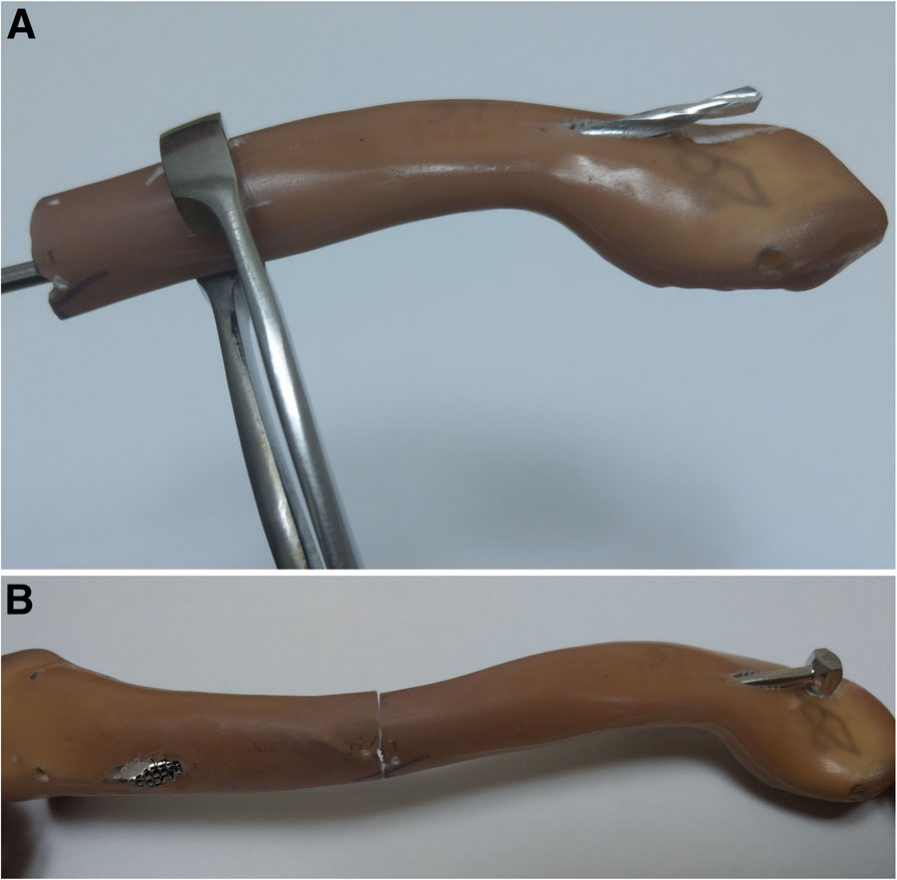
Experimental steps of fixation by Knowles pin. **a** Creating intramedullary path by 3.2 mm drill bit. **b** Insertion of 3.9 mm Knowles pin

For the Knowles pin group, a 3.9 mm diameter stainless Knowles pin (Smith & Nephew, Tuttlingen, Germany) was used as one intramedullary device (Fig. 1). Using a 3.2 mm drill bit, the intramedullary path was created by drilling from the osteotomy site of both the lateral and medial fragments through the intramedullary cavity. The Knowles pin was inserted into the intramedullary path from the lateral fragment until its tip fully penetrated the medial fragment (Fig. 2).

**Fig. 3:**
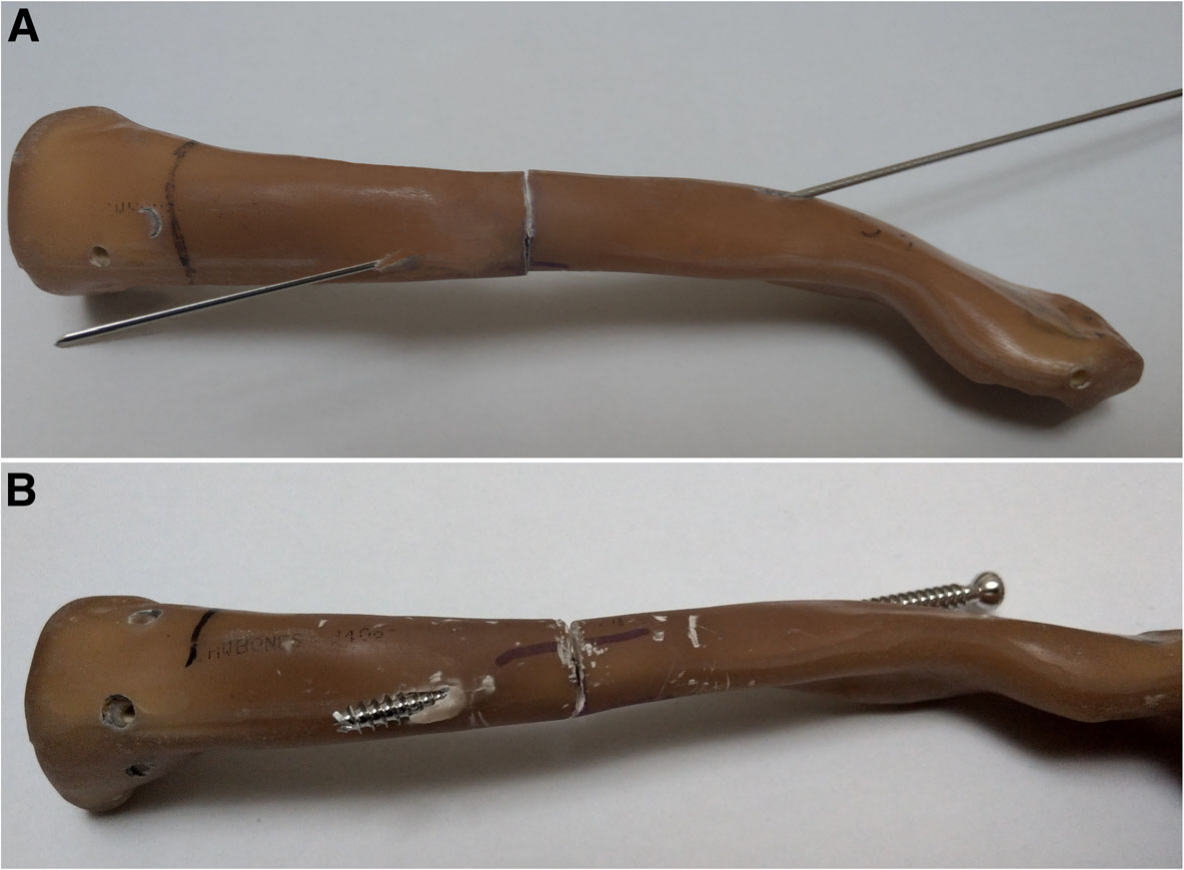
Experimental steps of fixation by cannulated screw. **a** 1.3 mm diameter Steinmann pin through the intramedullary canal. **b** Insertion of 4.5 mm cannulated screw

For the cannulated screw group, a 4.5 mm diameter stainless cannulated screw (Syntec, Chang Hua, Taiwan) was used as another intramedullary device (Fig. 1). A 1.3 mm diameter Steinmann pin was inserted through the intramedullary canal of both the medial and the lateral fragment. After tapping the intramedullary canal, the cannulated screw was inserted from lateral fragment through the intramedullary canal, with guidance by the pin until the screw tip fully penetrated the medial fragment (Fig. 3).

### Purchase length measurement of both type of intramedullary devices

Purchase length was defined as the engaged length of the intramedullary portion of the two intramedullary devices [10].

### Biomechanical test

**Fig. 4:**
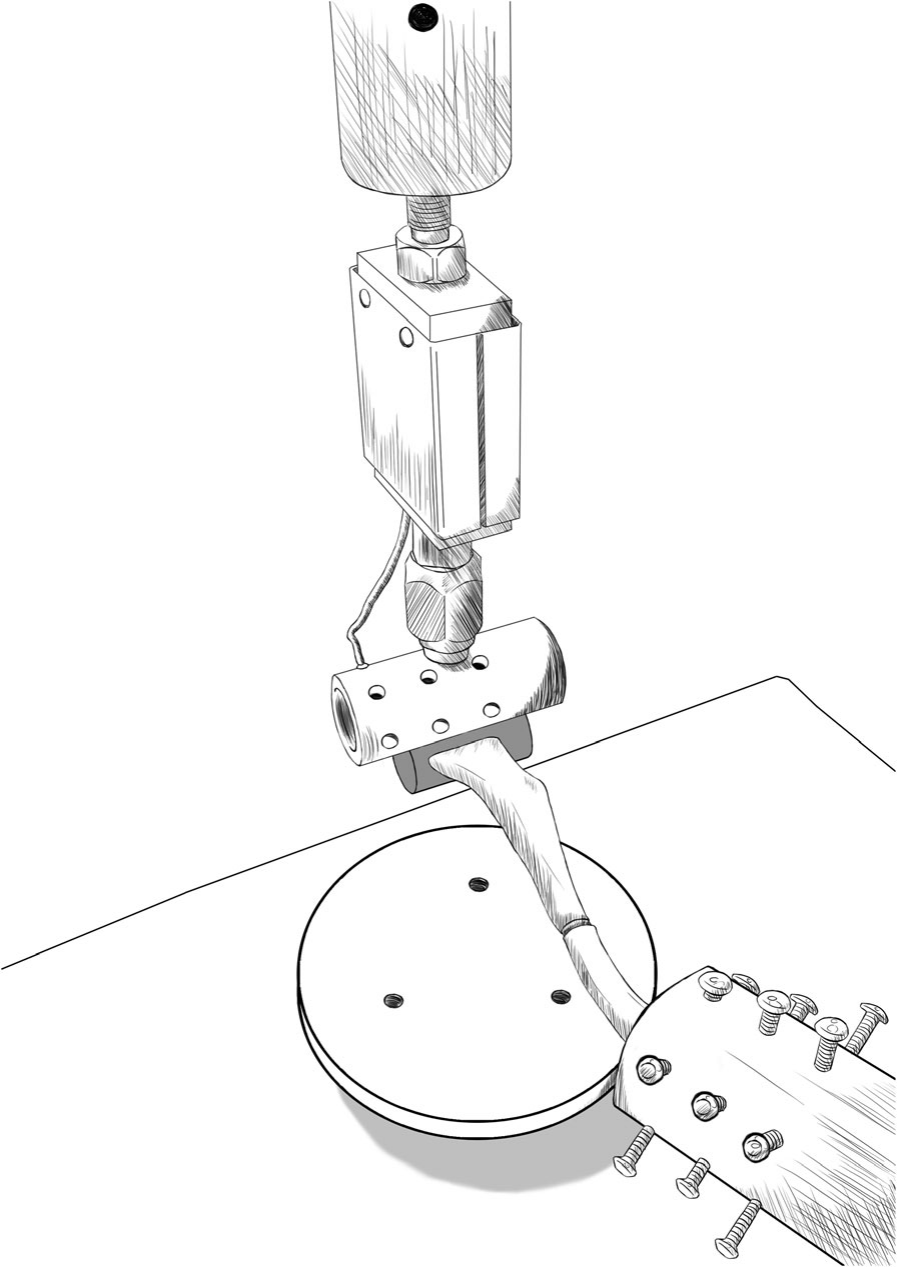
Representative illustration of cantilever bending testing

The most distal 2 cm of the lateral fragment was potted in polymethylmethacrylate within custom-made cylinders. The potting cylinder extended equally on both sides of the long axis of the clavicle so that the load did not induce a rotational moment [13]. A custom ring-jig with convergent fixation screws was constructed to fix the medial portion of the tested synthetic bone. The fixed portion of tested synthetic bone by concentric screws of the ring-jig was 4 cm. The position and orientation of the fixed specimen within the ring-jig was standardized. A servo-hydraulic materials testing system (MTS 858 Mini Bionix, MTS, Eden Prairie, MN) with a 200-kg load cell and flat-bottom actuator was used to create an inferior load in the lateral portion of each tested synthetic bone using the cantilever bending test (Fig. 4). Cantilever bending most closely replicates the forces experienced by the clavicle, with the relatively fixed sternoclavicular joint and the weight of the limb suspended from the lateral clavicle [14]. To precondition the specimen, a cyclic preload of 0 to 10 N was applied to each construct for 10 cycles at 1 Hz. Then, a downward force was applied to the lateral fragment in the displacement-control model at a rate of 12 mm/min until the construct failed. The load, actuator displacement, and time data were collected at a sampling rate of 10 Hz. The bending stiffness values were calculated using the linear portions of the load-displacement curve. A 0.2% offset method was used to calculate the yield point because the yield point could not be arbitrarily defined [14]. The load corresponding to the yield point was defined as the yield load. Maximum load was defined as the load that caused catastrophic failure in the tested synthetic bone fracture or implant failure resulting in the construct’s sudden inability to further withstand the applied load.

### Statistical analysis

In addition to the intramedullary purchase lengths of both intramedullary devices, which were analyzed by an independent t-test, all mechanical variables were analyzed using a multivariate analysis of variance to detect significant differences among the three treatment groups, using SPSS 20.0 (SPSS Inc., Chicago, IL). A Tukey post hoc test was applied, when necessary, to determine the specific differences between treatment groups.

## Results

The intramedullary purchase lengths of the Knowles pin and cannulated screw groups were 11.9 ± 0.5 and 8.0 ± 0.5 cm, respectively. The Knowles pin group had a significantly longer average intramedullary purchase length compared with that of the cannulated screw group (*p* < 0.0001) (Table 1).

**Table 1:** Summary of Data From the Cantilever Test (Mean ± SD)

	Treatment			ANOVA	Post hoc ANOVA *p* value		
	PLATE (*n* = 6)	PIN (n = 6)	SCREW (n = 6)	*p* value	PLATE vs PIN	PLATE vs SCREW	PIN vs SCREW
Stiffness(N/mm)	5.6 ± 0.9	3.1 ± 0.6	1.7 ± 0.4	< 0.0001	< 0.0001^a^	< 0.0001^b^	0.007^c^
Yield load(N)	83.3 ± 8.9	73.2 ± 10.5	43.4 ± 15.1	< 0.0001	0.332	< 0.0001^b^	0.001^c^
Maximum load(N)	134.6 ± 12.4	115.8 ± 16.8	77.8 ± 21.6	< 0.0001	0.181	< 0.0001^b^	0.005^c^
				t-test			
				*p* value			
Intramedullary Purchase Length (cm)	n/a	11.9 ± 0.5	8.0 ± 0.5	< 0.0001^c^			

^a^ Significant difference between plate and pin group; ^b^ Significant difference between plate and screw group; ^c^ Significant difference between pin and screw group

PLATE, reconstruction plate; PIN, Knowles pin; SCREW, cannulated screw

**Fig. 5:**
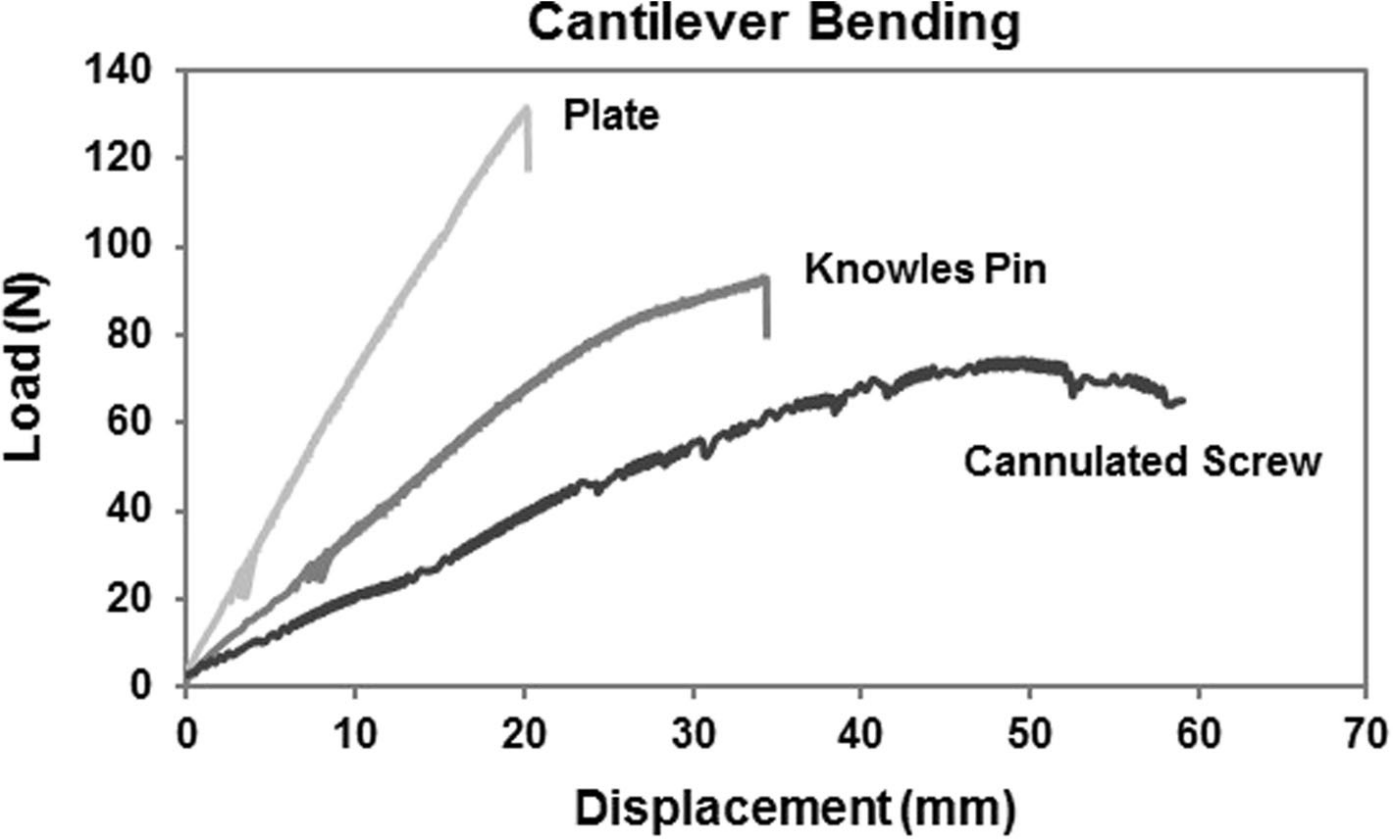
Example of cantilever bending load displacement data for representative trials from each implant construct

**Fig. 6:**
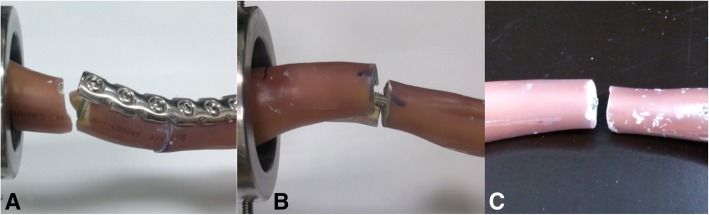
Example of failure modes for (**a**) reconstruction plate, (**b**) Knowles pin and (**c**) cannulated screw

The construct stiffness in the reconstruction plate group (5.6 ± 0.9 N/mm) was higher than that of the intramedullary devices; the Knowles pin group (3.1 ± 0.6 N/mm) provided a greater construct stiffness than did the cannulated screw group (1.7 ± 0.4 N/mm) (*p* = 0.007). The cannulated screw group had the lowest yield and maximum load of the three groups. The differences between the reconstruction plate group and the Knowles pin group did not reach statistical significance (Table 1). Figure 5 shows the typical load-displacement curves for the three groups. The typical failure modes differed between groups, as shown in Fig. 6. In the reconstruction plate group, all specimens failed at the most medial screw with a short-oblique fracture (Fig. 6a). In the Knowles pin group, all specimens failed over the medial fragment, with cracks and splits (Fig. 6b); only two of the six implants revealed very mild plastic angulation of less than 10 degrees. In the cannulated screw group, all implants broke over the osteotomy site (Fig. 6c).

## Discussion

This study investigated the mechanical effects of three types of fixation implant (reconstruction plate, Knowles pin, and cannulated screw) for a simulated midshaft transverse osteotomy using synthetic clavicles. The results showed that the reconstruction plate had the greatest bending stiffness, yield, and maximum load; by contrast, the cannulated screw group had the lowest values for all three measures. The Knowles pin group had a significantly longer intramedullary purchase length compared with the cannulated screw group. Thus, these results suggest that fixation of midshaft clavicle fractures with cannulated screws may lead to early failure due to inadequate mechanical strength and purchase length.

Various surgical options for midshaft clavicle fractures, including extramedullary and intramedullary devices, have proven effective [15]. Extramedullary devices provide more immediate stability, especially in a torsional test, than intramedullary devices in most biomechanical comparisons [14, 16, 17]. The advantages of intramedullary devices are smaller surgical incisions, less extensive dissections, load-sharing fixation that encourages copious callous formation, and easier removal than extramedullary devices [18]. Minimally invasive surgeries have gained attention in long bone fracture management not only because of the cosmetic effect but also because of fewer fracture union problems due to less soft tissue dissection. Many of these surgeries, including clavicle fixation, are conducted through hollow-designed implants. The guided implant facilitating procedures do not even need to open the fracture site [6, 7]. However, the hollow structures may reduce the mechanical strength of the implants [19]. Therefore, this study compared two common implants of midshaft clavicle fractures that are representative of traditional extramedullary and intramedullary devices, and added cannulated screws to represent the hollow-designed implant. The test protocol included only a cantilever test for bending stiffness but no torsional test because of the undoubted superior torsional stiffness of the extramedullary device. To the best of our knowledge, this is the first biomechanical study of the use of cannulated screws in midshaft clavicle fixation.

The results of this study revealed that the reconstruction plate has greater bending stiffness than the intramedullary devices. It is generally expected that intramedullary devices allow for more displacement, i.e., more flexibility, than extramedullary fixations. The result is compatible with a previous cyclic four-point bending test that used frozen clavicles to reveal more stability in plate fixation than in pin fixation during both displacement-control and load-control protocols [20]. The yield and maximum load were highest in the reconstruction plate group; these differences were not significant when compared with the Knowles pin group but were significant when compared with the cannulated screw group. The failure site of the reconstruction plate group was located at the most medial fixation screws, which experience the most stress, while the osteotomy site remained intact, a result consistent with previous studies [13, 14]. The Knowles pin group failed with a medial fragment split and crack with almost intact implants. The failure mode of both the reconstruction plate and the Knowles pin group revealed construct failure, i.e., failure occurred at the implant-bone interface with intact implants. However, the cannulated screw group, which had the lowest yield and maximum load, showed implant failure, i.e., failure at the osteotomy site with broken implants. This difference means that the cannulated screws have less mechanical strength than either the reconstruction plates or Knowles pins, and cannot provide adequate stability for fracture fixation.

The average intramedullary purchase length of the Knowles pin group was longer than that of the cannulated screw group due to its small diameter. The sigmoid shape of the clavicle allows a straight implant with a smaller diameter to purchase a longer intramedullary length than one with a larger diameter. The result is compatible with a previous study that compared 3.2 mm and 4.0 mm intramedullary pins and concluded that pins with a smaller diameter could achieve longer intramedullary engagement lengths [10]. From a biomechanical point of view, a longer leverage bending moment provides more stability for load-sharing intramedullary devices [21]; however, this goal can only be achieved using devices that have small diameters at the cost of reducing mechanical strength, which is influenced by the radius to its fourth power [19]. Hence, diameter selection presents a dilemma due to the compromise between intramedullary purchase length and mechanical strength in straight intramedullary clavicle devices with hollow structures because of the sigmoid shape of the clavicle intramedullary canal.

Criticisms concerning the evaluation of construct stiffness were mentioned in the previous studies. Without a quantifiable threshold measure for stiffness, an anticipated prediction can be drawn: “more and larger metal is stronger” [22]. In addition, biological factors, for example, the extend of intraoperative soft tissue dissection was not accounted in evaluating construct stiffness in the reviewed biomechanical studies [17]. An accomplished fact was observed from all mechanical studies that plating will provide greater torsional stiffness as compared to intramedullary fixation. Nevertheless, both implants deliver similar clinical successes [23]. It is assumed that postoperative temporary immobilization with the avoidance of shoulder abduction and flexion can minimize the effect of the lower torsional stiffness of the intramedullary device [24]. Postoperative pendulum exercises can be started as soon as possible to prevent shoulder joint stiffness without generating much rotational moment. However, bending movement to fracture is more difficult to avoid, even during pendulum exercise. Thus, the bending stiffness serves as a more clinically relevant measure than torsional stiffness in biomechanical tests.

This study revealed the inadequate mechanical strength of the cannulated implant for clavicle intramedullary fixation. Hollow designs facilitate minimally invasive surgeries by using a guide pin followed by the implant. However, the mechanical strength of hollow design implants should be evaluated thoroughly. The different failure modes (construct failure vs implant failure) indicated insufficiency mechanical strength of cannulated screws for clavicle midshaft fracture fixation. We think that the key design point of a guided intramedullary device should be the adequate purchase of the sigmoid intramedullary canal for a longer leverage bending moment and durable mechanical strength to prevent implant breakage. A new, well-designed clavicle nail is now available for midshaft clavicle fixation. Its greater flexibility allows the implant to adapt into the sigmoid shape of the clavicle, avoiding the compromise between the intramedullary purchase length and the mechanical strength of the straight implant. The Wavibody assembly converts the flexible nail into a rigid one. The locking mechanism provided by tail locking screw and Wavibody seems to be the solution of frequent implant backout of traditional IM devices due to lack of rotational stability [25]. A biomechanical test using synthetic clavicles showed the same strength as the plate in repairing midshaft clavicle fractures [13, 26]. But there are sporadic case reports discussing early failure of the new device [27, 28]. It is not appropriate to define the failure rate by these cases; however, further mechanical tests specifically focusing on the mechanical strength of the implants should be performed.

This study had two limitations. First, the comparison between the intramedullary devices contained two independent variables: implant diameter and structural difference. It is difficult to evaluate the contribution of each variable without regression analysis, which is not applicable in this study. Second, a simplified simulation of only one fracture model was used. The transverse osteotomy model was chosen for the current study because this simplified fracture pattern made it easier to control variability between the various repair constructs. However, more complicated and comminuted fractures are often encountered during clinical scenarios.

## Conclusion

Fixation of midshaft clavicle fractures with cannulated screws may lead to early failure due to inadequate mechanical strength. Ideal intramedullary clavicle devices should supply adequate intramedullary purchase lengths and mechanical strength.

## Abbreviations

AO: Arbeitsgemeinschaft für Osteosynthesefragen
n/a: Not applicable
